# Recurrent amnesia caused by early seizures after hippocampal infarction: a case report

**DOI:** 10.1186/s12883-021-02543-8

**Published:** 2022-01-10

**Authors:** Eckhard Schlemm, Tim Magnus, Leander D. Rimmele, Justine Münsterberg, Maxim Bester, Simon S. Kessner, Mathias Gelderblom, Christian Gerloff

**Affiliations:** 1 Klinik und Poliklinik für Neurologie, Kopf- und Neurozentrum, University Medical Center Hamburg-Eppendorf, Hamburg, Germany; 2 Klinik und Poliklinik für Neuroradiologische Diagnostik und Intervention, Zentrum für Radiologie und Endoskopie, University Medical Center Hamburg-Eppendorf, Hamburg, Germany

**Keywords:** Diffusion lesion, Ischemic stroke, Symptomatic seizure, Transient global amnesia, Transient epileptic amnesia

## Abstract

**Background:**

We report the case of a patient with recurrent episodes of disturbed memory suggestive of transient epileptic amnesia, and a focal hippocampal lesion typically associated with transient global amnesia. We argue how careful consideration of clinical, electrophysiological and imaging findings can resolve this apparent contradiction and lead to a diagnosis of early symptomatic post-stroke seizures that links brain structure to function in a new, clinically relevant way.

**Case presentation:**

A 70-year-old patient was identified in clinical practice in our tertiary care centre and was evaluated clinically as well as by repeated electroencephalography and magnetic resonance imaging. The presenting complaint were recurrent episodes of short-term memory disturbance which manifested as isolated anterograde amnesia on neurocognitive evaluation. EEG and MRI revealed predominantly right frontotemporal spikes and a punctate diffusion-restricted lesion in the left hippocampus, respectively. Both symptoms and EEG changes subsided under anticonvulsant treatment with levetiracetam.

**Conclusions:**

Our report contributes to the current discussion of clinical challenges in the differential diagnosis of transient memory disturbance. It suggests that focal diffusion-restricted hippocampal lesions, as seen in TGA, might be ischemic and thus highlights the importance of considering post-stroke seizures as a possible cause of transient memory disturbance.

## Background

Differential diagnoses for atraumatic temporary memory loss include cerebral ischemia, transient epileptic amnesia (TEA), and transient global amnesia (TGA). Diagnostic criteria for TEA are recurrent short episodes of disturbed memory; sparing of other cognitive domains; and clinical or electroencephalographic (EEG) evidence of seizure activity or response to anticonvulsant medication [1]. Memory impairment in TGA, in contrast, lasts longer, rarely recurs, and is not believed to be of epileptic origin. Diffusion abnormalities on magnetic resonance imaging (MRI) are rarely observed in TEA, tend to be diffuse, and likely represent a postictal phenomenon [2]. In TGA, punctate hippocampal foci of restricted diffusion develop in up to over 80% of patients [3]. We report the first case of a patient fulfilling the clinical criteria for TEA in the presence of a focal hippocampal lesion on diffusion imaging, and argue that, in this constellation, early post-stroke seizures are the most likely diagnosis.

## Case report

**Fig. 1:**
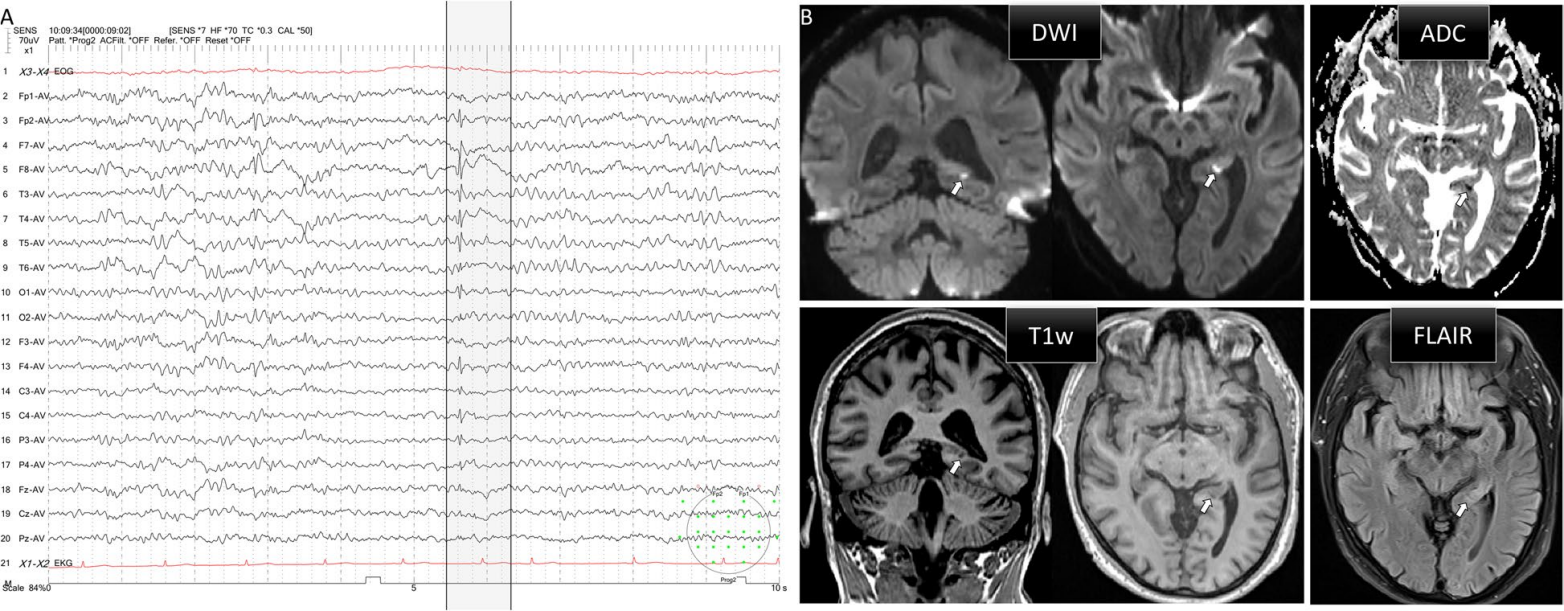
**A** Scalp electroencephalography recording on the day of admission 20 h after most recent amnesic episode. Highlighted is a right frontotemporal spike followed by a slow wave. Four seconds prior to this prominent transient, there is a beta wave followed by a low-amplitude spike in the left temporal region **B** Magnetic resonance imaging 48 h after first amnesic episode. White arrows indicate a punctate focus of restricted diffusion and low ADC signal (top row) in the tail of the left hippocampus. Signal intensity in the lesion is low on T_1_-weighted and high on T_2_-weighted (FLAIR) sequences (bottom row)

A 70-year-old man presented to the emergency department after a 30-min episode of anterograde amnesia and disorientation. A similar episode had occurred the previous day. Past medical history included hypertension; paroxysmal atrial fibrillation for which he took apixaban 2 × 5 mg daily; mitral valve insufficiency; and a recent urinary tract infection treated with ciprofloxacin. On examination, he was fully oriented without focal neurological deficits. Montreal Cognitive Assessment (MoCA) revealed a persistent anterograde amnesia with no further cognitive impairments (score 26/30). Routine blood tests and computed tomography of the head were unremarkable. Initial electroencephalography after spontaneous resolution of symptoms showed intermittent right frontotemporal spikes followed by slow waves. They were preceded by intermittent beta waves and low-amplitude spikes in the left temporal region (Fig. 1A); diffusion-weighted magnetic resonance imaging (DWI) 48 h after first symptoms revealed a punctate hyperintense area with reduced ADC signal in the lateral part of the tail of the left hippocampus; the lesion was T_1_-hypointense and rather hyperintense in FLAIR images (Fig. 1B). After admission, there was complete spontaneous remission of symptoms before another self-limiting episode of amnesia and disorientation was observed. A repeat EEG during the attack showed that the spikes had regressed over time, in line with the interpretation that the symptoms might, in part, have been postictal. After the patient was started on levetiracetam 750 mg twice daily, no further symptoms were experienced and a repeat EEG on day 4 did not show any epileptiform discharges. Further diagnostic workup included a lumbar puncture as well as blood tests, neurosonography and transthoracic echocardiography to screen for common causes of ischemic stroke; beyond dyslipidaemia no relevant new abnormalities were found and the patient was discharged on levetiracetam and a statin. On follow-up after 4 weeks on this medication, no further episodes of amnesia had occurred. A repeat MRI after 9 weeks under continued medication showed complete resolution of the lesion on DWI and no residual FLAIR changes.

## Discussion

Despite their definition as distinct phenotypes of transient memory disturbance, differentiation between TGA and TEA can be challenging. When clinical features are inconclusive, diffusion imaging and EEG may aid diagnosis. Their usefulness is limited, however, by the facts that the appearance of characteristic lesions in the hippocampus in TGA is often delayed [3] and that interictal EEG recordings can be normal or unspecific in up to two thirds of TEA patients [1].

Conflicting positive findings, on the other hand, also present challenges. Arguing that the lesion in our patient was too circumscribed to be a postictal phenomenon, and ignoring the possibility of a coincidental finding, it follows that it played a causal role for his symptoms. While TEA is usually idiopathic, a small number of symptomatic cases caused by focal lesions in the temporal lobe have been reported previously [1]. Diffusion lesions in TGA are typically small and may occur in all parts of the hippocampus with a preference for its body [4]. While the underlying pathology remains obscure, their epileptogenic potential seems limited. Indeed, genuinely epileptiform discharges have rarely been recorded in TGA patients [5], and longitudinal studies showing complete resolution of the diffusion lesion after 4 months suggest a minor impact on hippocampal structure [6]. On the other hand, both symptomatic seizures after brain infarction, and stroke as a cause of isolated amnesia are well recognized.

In the presented case, imaging was consistent with both a recent ischemic infarct and a TGA lesion, although the posterior location in the hippocampus and low T_1_-intensity are unusual for TGA. Considering the high frequency of recurrent amnesic episodes and consistent EEG changes thus led us to the diagnosis of early post-stroke seizures.

We suggest that this differential be considered in cases of transient amnesia with a suspicious lesion in the hippocampus, especially if symptoms recur or are associated with epileptiform discharges on EEG, as it might inform secondary prevention and influence the medical management of the patient.

## Data Availability

Data sharing is not applicable to this article as no datasets were generated or analysed during the current study.
